# Automated Fragmentation QM/MM Calculation of NMR Chemical Shifts for Protein-Ligand Complexes

**DOI:** 10.3389/fchem.2018.00150

**Published:** 2018-05-08

**Authors:** Xinsheng Jin, Tong Zhu, John Z. H. Zhang, Xiao He

**Affiliations:** ^1^State Key Laboratory of Precision Spectroscopy, School of Chemistry and Molecular Engineering, Shanghai Engineering Research Center of Molecular Therapeutics and New Drug Development, East China Normal University, Shanghai, China; ^2^NYU-ECNU Center for Computational Chemistry at NYU Shanghai, Shanghai, China; ^3^Department of Chemistry, New York University, New York, NY, United States; ^4^National Engineering Research Centre for Nanotechnology, Shanghai, China

**Keywords:** AF-QMMM, NMR chemical shift, protein-ligand binding, scoring function, structure prediction

## Abstract

In this study, the automated fragmentation quantum mechanics/molecular mechanics (AF-QM/MM) method was applied for NMR chemical shift calculations of protein-ligand complexes. In the AF-QM/MM approach, the protein binding pocket is automatically divided into capped fragments (within ~200 atoms) for density functional theory (DFT) calculations of NMR chemical shifts. Meanwhile, the solvent effect was also included using the Poission-Boltzmann (PB) model, which properly accounts for the electrostatic polarization effect from the solvent for protein-ligand complexes. The NMR chemical shifts of neocarzinostatin (NCS)-chromophore binding complex calculated by AF-QM/MM accurately reproduce the large-sized system results. The ^1^H chemical shift perturbations (CSP) between apo-NCS and holo-NCS predicted by AF-QM/MM are also in excellent agreement with experimental results. Furthermore, the DFT calculated chemical shifts of the chromophore and residues in the NCS binding pocket can be utilized as molecular probes to identify the correct ligand binding conformation. By combining the CSP of the atoms in the binding pocket with the Glide scoring function, the new scoring function can accurately distinguish the native ligand pose from decoy structures. Therefore, the AF-QM/MM approach provides an accurate and efficient platform for protein-ligand binding structure prediction based on NMR derived information.

## Introduction

Structure-based computational methods are useful tools for analyzing the binding modes and affinities for protein-ligand complexes (Grinter and Zou, 2014). With the development of X-ray crystallography and nuclear magnetic resonance (NMR) technology, more than 100,000 high resolution three-dimensional protein structures have been determined, which is helpful for finding lead compounds and therapeutic targets (Ferreira et al., 2015). As compared to experimental methods, computational approaches, such as molecular docking, are the fast and efficient ways for drug discovery. In molecular docking programs, the scoring functions are used to approximate the binding free energy and hence to rank the simulated decoy poses (Sliwoski et al., 2014). Most scoring functions are roughly derived from force-field-based (Morris et al., 1998; Englebienne and Moitessier, 2009), empirical (Eldridge et al., 1997; Murray et al., 1998) or knowledge-based potentials (Gohlke et al., 2000; Huang and Zou, 2006). However, based on parameterized functions, these scoring methods are usually not accurate enough to differentiate the experimental structure from the docked decoy structures, and sometimes the rankings from different software suites may be inconsistent (Śledź and Caflisch, 2018). For solving this problem, much effort has been devoted to the development of docking methods by introducing the experimental structural information or quantum mechanical (QM) calculations for scoring the native and predicted poses (Mohan et al., 2005; Grinter and Zou, 2014; Adeniyi and Soliman, 2017).

The chemical shift is one of the most effective and precise NMR parameters in reflecting the local chemical environment around the atom, which plays an important role in structure determination and refinement (Zhu et al., 2014; Bratholm and Jensen, 2017). For NMR chemical shift calculations, the empirical chemical shift prediction softwares include ShiftS (Xu and Case, 2001; Moon and Case, 2007), ShiftX (Neal et al., 2003), ShiftX2 (Han et al., 2011), CamShift (Robustelli et al., 2010), PROSHIFT (Meiler, 2003; Meiler and Baker, 2003), SHIFTCALC (Williamson and Craven, 2009), ProCS (Christensen et al., 2014; Bratholm and Jensen, 2017), CheShift (Vila et al., 2009; Garay et al., 2014) and Sparta+ (Shen and Bax, 2010). These empirical methods are fast in computational speed, and have been successful in predicting backbone chemical shifts. As the empirical formulas for these models are derived from fitting the experimental or QM calculated chemical shift database and the high-quality structures, these models are not well suited for accurate prediction of NMR chemical shifts for some complex systems such as protein-ligand complexes, non-standard protein residues or non-canonical base pairs in nucleic acid systems (Swails et al., 2015). The quantum mechanical chemical shift calculations are in principle able to predict the NMR chemical shifts for any complex systems (Lodewyk et al., 2012; Hartman and Beran, 2014; Merz, 2014). For protein NMR chemical shift calculations, Cui and Karplus had proposed a very effective QM/MM approach (Cui and Karplus, 2000), Gao et al. developed fragment molecular orbital (FMO) method (Gao et al., 2007, 2010), Exner and coworkers utilized the adjustable density matrix assembler (ADMA) approach (Frank et al., 2012; Victora et al., 2014), Tan and Bettens developed the combined fragmentation method (CFM) (Tan and Bettens, 2013), and He and coworkers developed the automated fragmentation quantum mechanics/molecular mechanics (AF-QM/MM) method (He et al., 2009, 2014; Zhu et al., 2012, 2013, 2014, 2015; Swails et al., 2015; Jin et al., 2016) These fragment-based QM methods have been successfully applied for NMR chemical shift calculation of proteins and nucleic acids.

On the basis that chemical shifts or chemical shift perturbations (CSP) are sensitive to the variations of chemical environment, these parameters are quite suitable for structure determination (Case, 1998; Cavalli et al., 2007; Shen and Bax, 2015). Many NMR-based methods have been developed for prediction of protein-ligand binding modes (Medek et al., 2000; Cioffi et al., 2008; Riedinger et al., 2008; Aguirre et al., 2014). McCoy and Wyss utilized proton CSP data, induced by aromatic ring current effect in the ligands, to locate the ring position of docking structures (McCoy and Wyss, 2002). Recently, Ten Brink et al. compared the experimental and simulated CSPs to verify protein conformational changes and developed the CSP-based docking method (Ten Brink et al., 2015). Merz et al. developed CSP-based scoring functions to determine the binding poses for protein-ligand complexes (Wang et al., 2007; Yu et al., 2017). However, most of these scoring functions only calculate the proton chemical shift on proteins. The scoring functions could be more efficient by taking ligand ^1^H chemical shifts into consideration.

In this work, we applied the AF-QM/MM approach for NMR chemical shift calculations on protein-ligand binding complexes. Based on DFT calculations, the ^1^H chemical shifts on both protein and ligand are available for structure determination and improving the scoring functions. In the framework of the AF-QM/MM approach, the ligand is also divided into smaller fragments, and hence it significantly reduced the computational cost for ^1^H NMR chemical shift calculation on the large ligand. In this study, the neocarzinostatin (NCS) protein is selected as the test case for the AF-QM/MM method because of its importance in cancer therapy. NCS has experimental chemical shifts for both apo and holo forms (Myers et al., 1988; Mohanty et al., 1994; Schaus et al., 2001; Takashima et al., 2005; Wang and Merz, 2010). Furthermore, by comparing the AF-QM/MM calculated chemical shifts with experiment data, a chemical shift based scoring function was developed to rank the native and predicted protein-ligand binding poses.

This paper is organized as follows: first, a benchmark test was performed using the AF-QM/MM method for NMR chemical shift calculations of protein-ligand complex. The computed results are compared to the large-sized system NMR chemical shift calculations. Subsequently, AF-QM/MM calculated chemical shifts are compared with the experimental results for both apo and holo NCS structures. Next, the performance of chemical shift based and conventional energy based scoring functions on the rankings of predicted protein-ligand binding poses is discussed. Finally, the hybrid scoring function, that combines the calculated NMR chemical shifts and binding energy, is applied to rank the experimental structure and other docked binding poses.

## Computational approaches

### Structure preparation

The X-ray structures of apo and holo NCS were download from the Protein Data Bank (PDB ids: 1NOA and 1NCO, respectively). The experimental chemical shift data of apo protein and chromophore were obtained from previous studies (Myers et al., 1988; Mohanty et al., 1994). The holo NCS experiment chemical shifts were downloaded from Biological Magnetic Resonance Data Bank (BMRB entry: 5969). The structure minimization of the protein X-ray structures was performed using the AMBER12 program (Case et al., 2012) with the ff99SB force field. The apo and holo NCS structures were solvated in a truncated octahedral periodic box of TIP3P water molecules with each side at least 10 Å from the nearest solute atom (Jorgensen and Jenson, 1998). After the entire system was neutralized with counter ions, 1,000 steps of steepest descent algorithm following with 4,000 steps of conjugate gradient method were used to remove the improper contacts of the system. For obtaining the force field parameters of the ligand, the general AMBER force field (GAFF) (Wang et al., 2004) and AM1-bond charge correlations (AM1-BCC) charge model were utilized for the ligand (Jakalian et al., 2002). The molecular docking was performed using the Glide module in the Schrödinger program (Friesner et al., 2004; Halgren et al., 2004). The scoring function used in this study was Glide XP. In this study, the protein structure was fixed when the ligand was docked into the binding site. Therefore, we did not include the flexibility of the protein during molecular docking. Based on the optimized experimental structure using the molecular force field, 38 docking poses of the ligand predicted by Glide, whose RMSDs range from 1.5 to 10.5 Å with reference to the native position, were selected for subsequent chemical shift calculations at the QM level.

### The AF-QM/MM method for NMR chemical shift calculation of the protein-ligand complex

**Figure 1:**
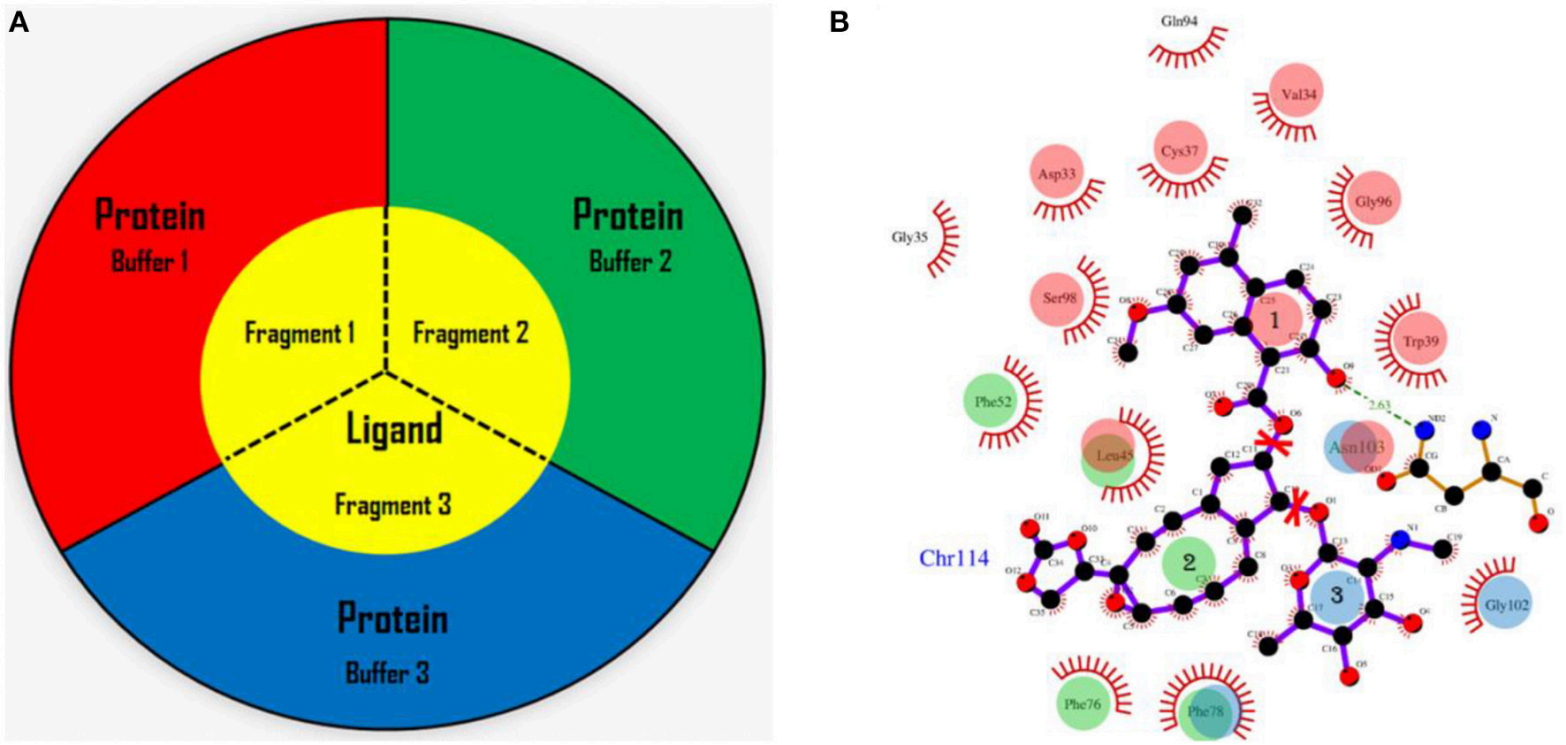
The fragmentation scheme for the ligand in AF-QM/MM. **(A)** The ligand is divided into three fragments. Each fragment of the ligand is taken as the core region. The buffer region contains remaining part of the ligand and protein residues within the certain distance threshold from the core region (see the text for more details). The core and buffer regions are calculated at the QM level while rest of the system are described by embedding charges. **(B)** The definition of each core region of the chromophore. Fragment 1: the naphthoate group; fragment 2: the enediyne ring part; fragment 3: the aminosugar group. The buffer region has the same color as the core region.

In the AF-QM/MM approach (He et al., 2009, 2014; Zhu et al., 2012, 2013; Swails et al., 2015; Jin et al., 2016), the apo protein is divided into individual residue by cutting through the peptide bonds. The number of fragments is the same as the number of residues in the protein. Each fragment contains a core region (each amino acid) in the protein, and the buffer region which contains the nearby residues surrounding the core region. Both the core and buffer regions are treated with quantum mechanics (QM) while the residues outside the buffer region are described by molecular mechanics (MM). The details for the definition of the buffer regions are described in our previous work (He et al., 2009; Zhu et al., 2012, 2013). For the holo protein studied in this work, we developed the fragmentation scheme for the ligand and its surrounding protein residues. As shown in Figure 1, we also divided the chromophore into three parts by cutting the C-O single bond. Fragment 1 contains the naphthoate group, fragment 2 includes the enediyne ring and fragment 3 has the aminosugar group, respectively. For each fragment of the ligand (taken as each core region), the rest part of the ligand and the protein residues surrounding the core region (each fragment of the ligand) are taken as the buffer region (see Figure 1). The distance criteria for selecting the buffer region for each fragment of the ligand is the same as that for each residue in the protein.

In this study, we adopt the following distance-dependent criteria to include residues within the buffer region of each core region for ligand: (1) if a heavy atom of the residue is less than 3.5 Å away from any atom in the core region, (2) if the distance of a hydrogen atom of the residue is less than 3.0 Å away from any atom in the core region. The cutoff enables a sufficient size of the buffer region for the convergence of chemical shift calculations on the core region (Flaig et al., 2012). The remaining residues beyond the buffer region are described by embedding charges to account for the electrostatic field outside the QM region. The protein charges were obtained directly from the ff99SB force field. For assigning the buffer region for each residue (where each residue is defined as the core region), the ligand is treated as a whole molecule (non-fragmented), and the definition of the buffer region follows the same criteria as the apo protein. The dangling bonds are capped with hydrogen atoms for constructing the closed-shell fragment.

The fragment QM calculations were carried out in parallel at the B3LYP/6-31G^**^ level. All QM calculations were performed using the Gaussian 09 package (Frisch et al., 2009). Only the NMR isotropic shieldings of the core region atoms were collected from each fragment QM calculation. The ^1^H chemical shifts are obtained by referencing to that of the tetramethylsilane (TMS) at the same computational level, which is 31.66 ppm. The implicit solvation model was applied to approximate the solvent effect. The protein charge distribution polarizes the dielectric solution and creates a reaction filed to act back on the solute until equilibrium is reached. The reaction field acting on the solute can be effectively represented by the induced charges on the cavity surface. In this work, the surface charges are calculated by the Poisson-Boltzmann (PB) model using the Delphi program (Rocchia et al., 2001). The set of point charges of the MM environment and on the molecular surface, which represents the reaction field, are used as the background charges in the QM calculation. Because the computational cost of QM chemical shift calculations will be dramatically increased on multiple configurations when the conformational sampling effect is taken into account. In this study, the optimized X-ray structure using molecular force field was taken as a representative configuration for the ensemble averaging structure.

### Scoring functions

**Figure 2:**
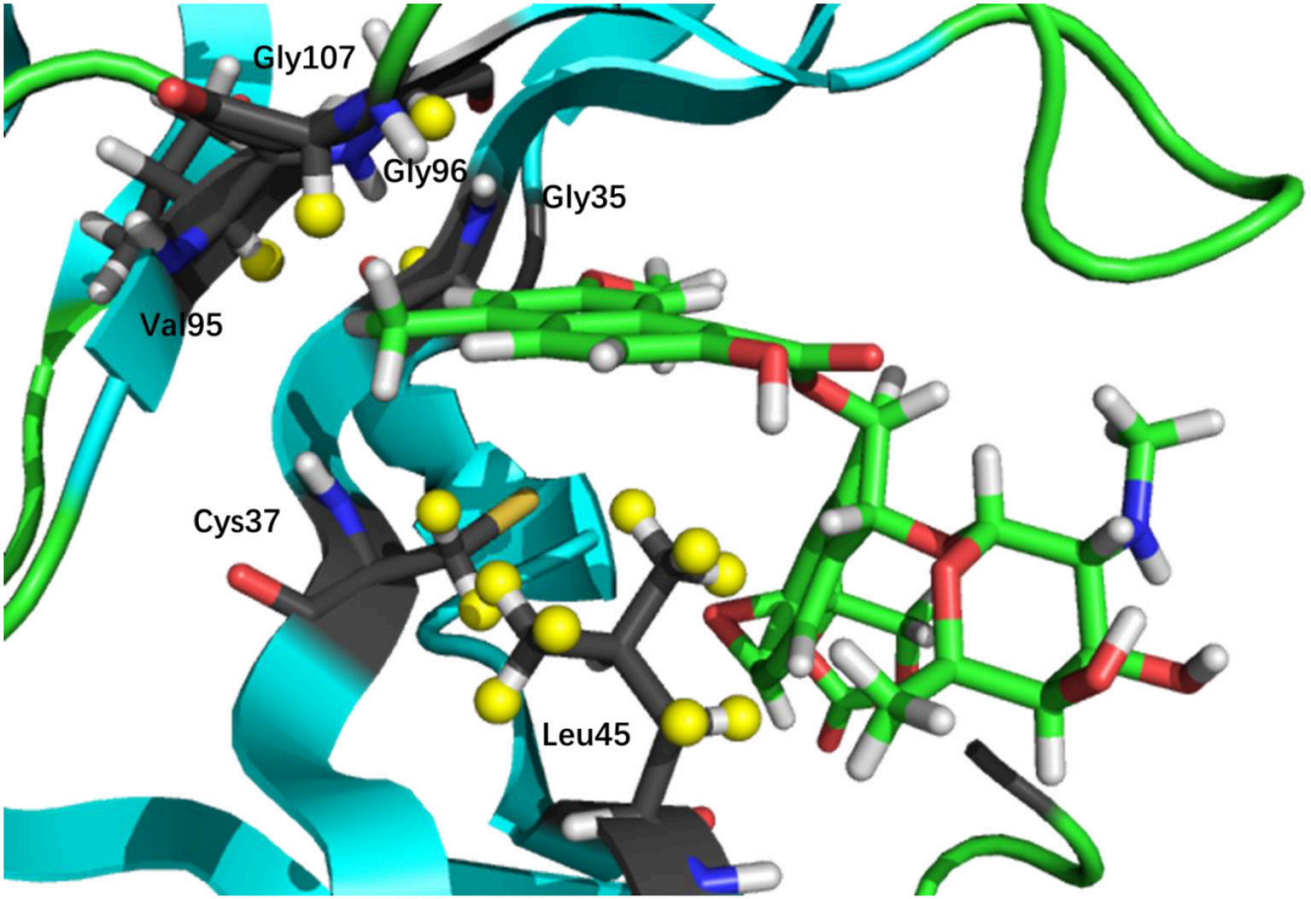
3D structure of the binding pocket. The nonpolar hydrogen atoms on the ligand, and protein protons whose CSP values (between bound and unbound complexes) are greater than 0.5 ppm (denoted in yellow) are chosen in the chemical shift based scoring functions (namely, *CS*_score_ and *CS*_Gscore_).

To differentiate the native protein-ligand binding structure from decoy poses, here we propose a scoring function based on NMR chemical shifts (CS_score_), which is simply the root-mean-square deviation (RMSD) of computed chemical shifts with reference to the experimental values,

(1) $$\begin{array}{r} \text{CSscore}=\sqrt{\frac{\sum_{i=1}^{N}\left(\delta_{\text{H}}^{i}-\delta_{\text{exp}}^{i}\right)}{N}} \end{array}$$

where $\delta_{\text{H}}^{i}$ is the chemical shift of *i*th hydrogen atom on the ligand and nearby residues, and $\delta_{\exp}^{i}$ is the experimental chemical shift of the corresponding atom in the native complex (holo NCS). *N* is the number of atoms whose chemical shifts were selected as molecular probe to characterize the NCS-chromophore binding structure. In this study, *N* was set to 31 for holo NCS, 21 of which are non-amide protons on the chromophore, and the other 10 hydrogen atoms are those with experimental chemical shift perturbations (CSP, between the bound and unbound complexes) greater than 0.5 ppm from residues in the binding site of the protein (see Figure 2 and Table S1 of the Supplementary Materials).

It is worth noting that, in Equation (1), we could also add the chemical shifts of the hydrogen atoms that experimentally do not change upon ligand binding. A false docking pose may cause significant deviations in CSPs for those hydrogen atoms. However, there are many of such protons on the residues in the binding pocket, which will average out the final score to make the scoring function incapable of distinguishing the native structure from the decoy sets. Protons with experimental perturbations greater than 0.5 ppm are more sensitive to the binding pose, therefore we took those atoms into account in the scoring function. Furthermore, although NMR chemical shifts of amide protons are also very sensitive to the local chemical environment of the binding pocket, these atoms were excluded owing to the lack of experimental data.

The second scoring function (*CS*_Gscore_) we propose here, is a linear combination of CS_score_ and Glide score,

(2) $$\begin{array}{r} \text{C}{\text{S}}_{\text{Gscore}}=\text{CSscore}+\alpha \text{Glide Score} \end{array}$$

where α is a weighting factor. In this study, the ranges of CSscore and Glide Score are 0.42~2.90 and −10.96~10.98 (see Table S2 of the Supplementary Materials), respectively, and thus we choose α = (2.90–0.42)/(10.98–(−10.96)) ≈ 0.1. By adding the Glide score to CS_score_, the unphysical structure with an unfavorable binding energy will be avoided. Since both the CS_score_ and Glide score will be smaller as the docking pose gets closer to the experimental structure, the native docking pose will give the lowest CS_Gscore_ value.

## Results and discussion

### Benchmark test of AF-QM/MM on the native NCS-chromophore binding complex

**Figure 3:**
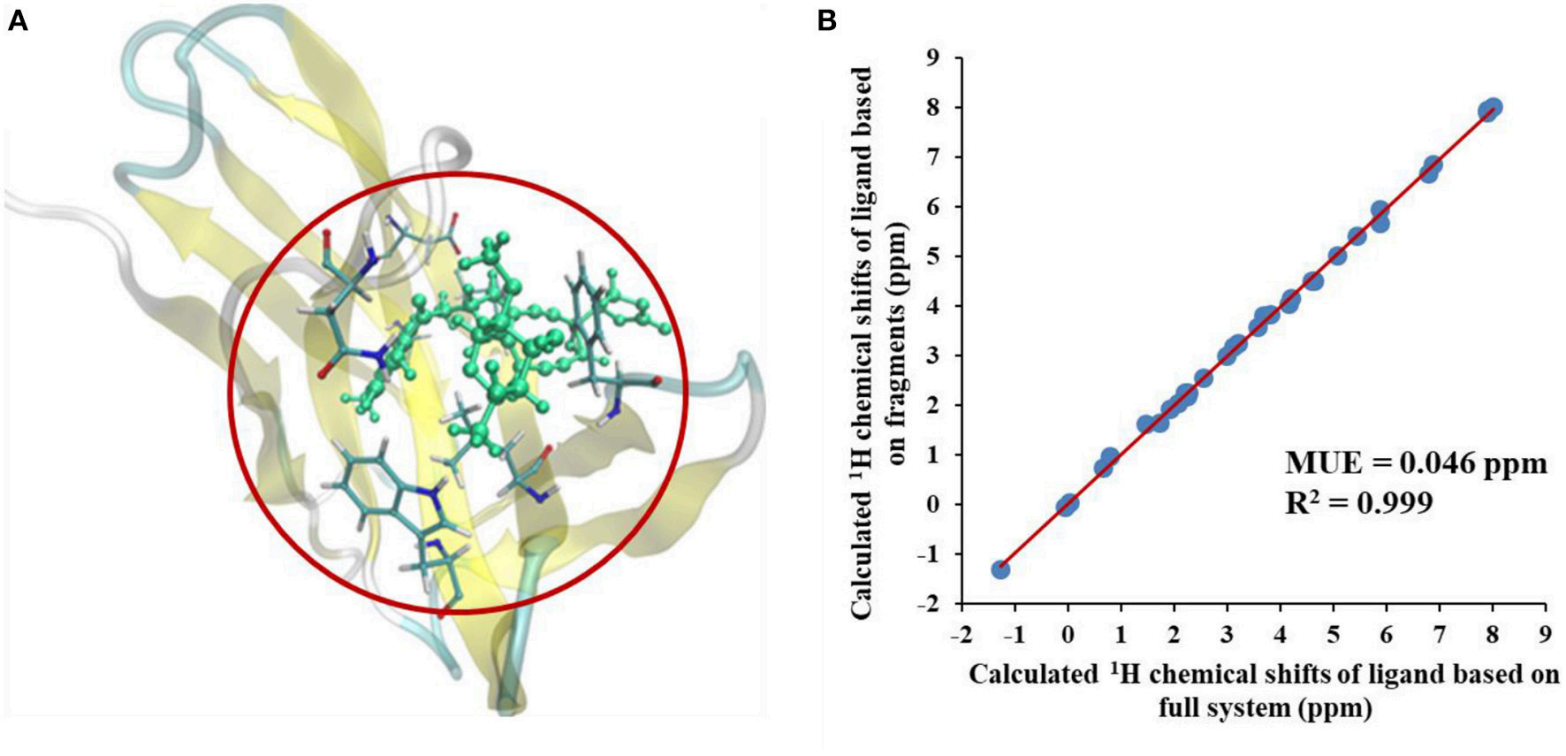
**(A)** The large-sized system contains the ligand and its buffer region (around 460 atoms) in holo NCS. **(B)** Comparison of calculated ^1^H chemical shifts between large-sized system calculation and the AF-QM/MM approach. “full system” denotes the large-sized system calculation.

We first compared the calculated chemical shifts on the chromophore between AF-QM/MM and large-sized system calculations. Because the holo NCS contains more than 1500 atoms and was too large to perform full system QM calculations, we alternatively used the entire ligand and its buffer region for large-sized QM calculation. The other atoms beyond the buffer region are taken as background charges, and the PB surface charges for the entire complex are also placed to approximate the implicit solvent. The computed chemical shifts from such a model system (around 460 atoms in the QM region) are taken as the reference values. Here, we only compare the chemical shifts on the ligand between AF-QM/MM calculation and large-sized system calculation. As shown in Figure 3, the ^1^H chemical shifts on the ligand calculated by the AF-QM/MM method (where the ligand is divided into three parts) are in good agreement with large-sized system calculation. The mean unsigned error (MUE) between AF-QM/MM results and chemical shifts from the large-sized system calculation is 0.046 ppm, and the RMSD between them is 0.051 ppm. The results demonstrate that the AF-QM/MM approach can accurately reproduce the large-sized system calculation. Furthermore, at the DFT level, the total computational cost was reduced by 36%, from 5,601 min (CPU time) by the large-sized system calculation to 3,585 min by dividing the ligand into 3 fragments. In addition, the 3 fragment-based QM/MM calculations were carried out in parallel. Therefore, the computational wall time could be further reduced by approximately 2/3.

**Figure 4:**
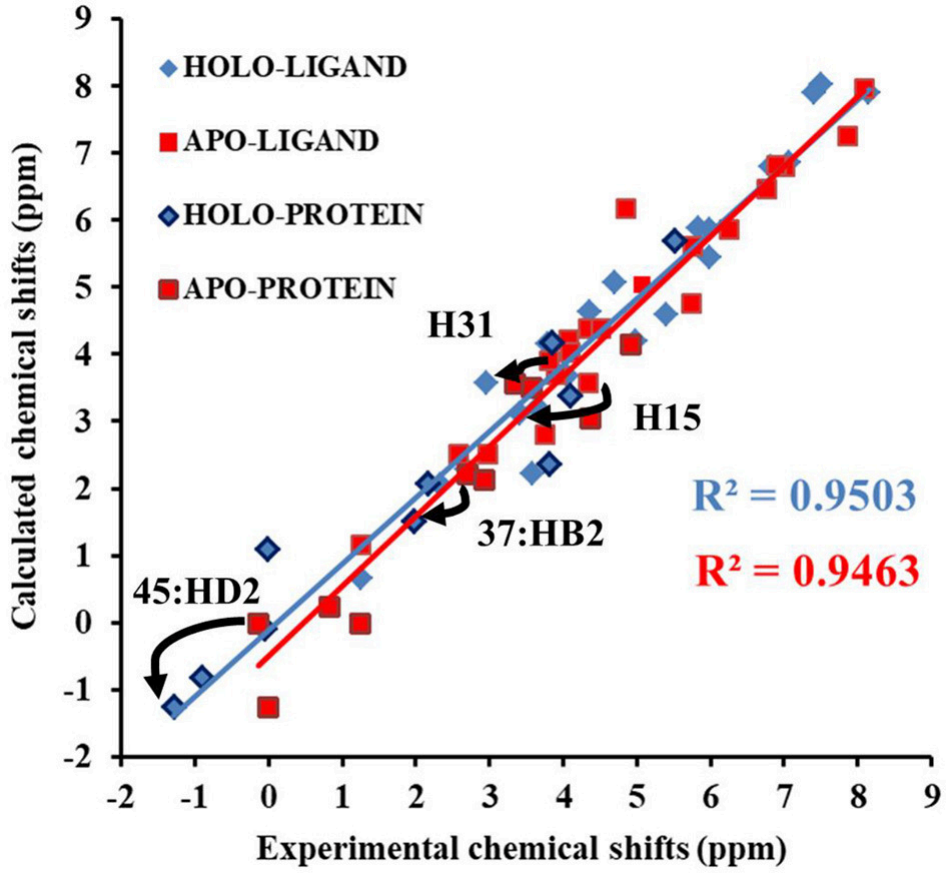
Comparison of the AF-QM/MM results and experimental ^1^H chemical shifts (31 protons in the binding pocket, as shown in Figure 2) for both apo and holo NCS.

Next, we compare the calculated chemical shifts for apo and holo NCS (31 protons in the binding pocket, as shown in Figure 2) with the experimental values. In this benchmark test, the AF-QM/MM results correlate well with the experiment (see Figure 4). For the bound complex, the MUE between the calculated and experimental chemical shifts is 0.44 ppm, and the RMSD is 0.57 ppm. For the unbound protein and ligand, the MUE and RMSD between calculated and experimental chemical shifts are 0.45 and 0.62 ppm, respectively.

**Figure 5:**
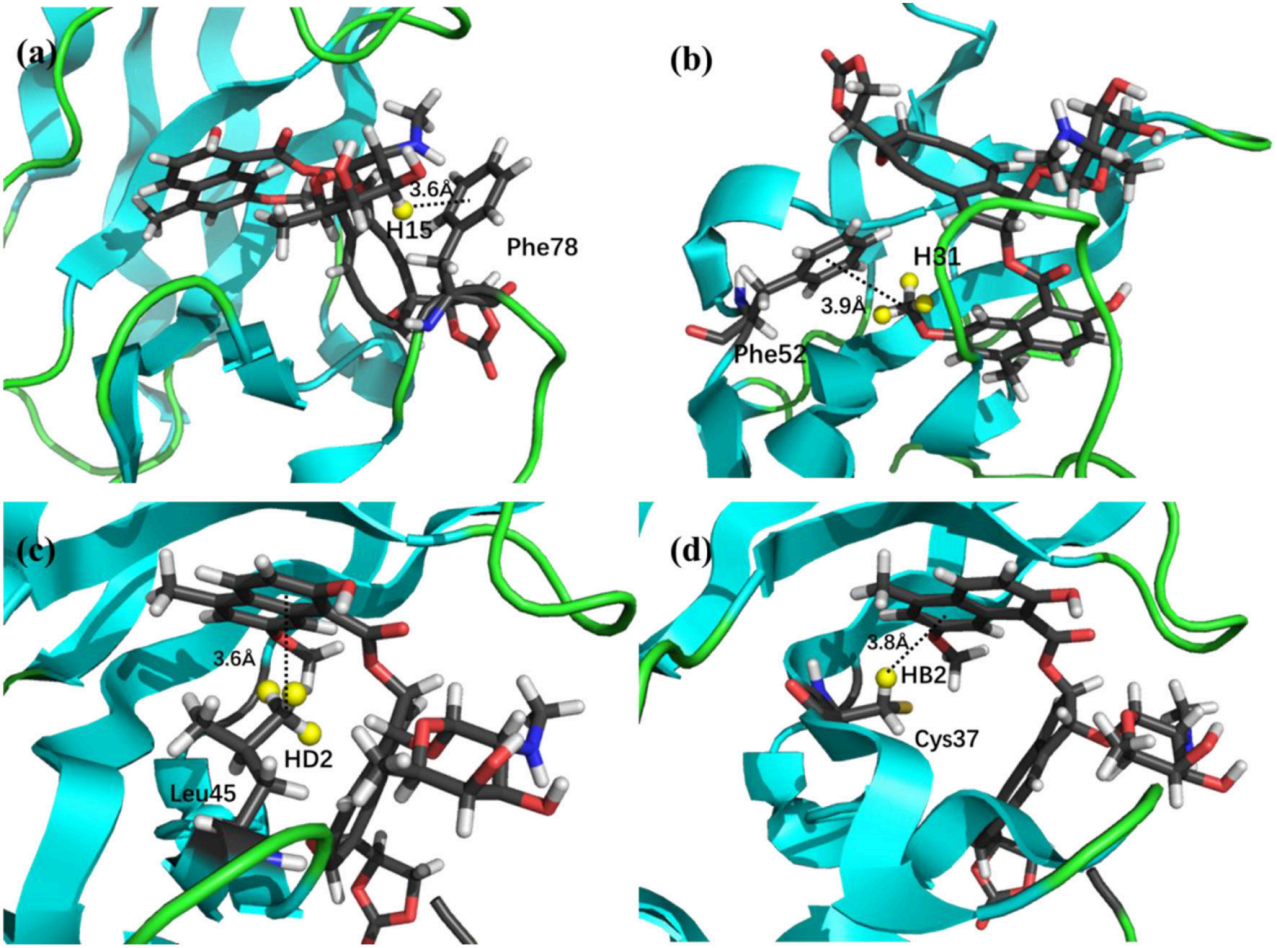
X-ray structure of the holo NCS. The CSPs for protons of H15 **(a)** and H31 **(b)** in the chromophore, Leu45:HD2 **(c)** and Cys37:HB2 **(d)** in the NCS, are significantly influenced by the ring current effect.

The prediction of chemical shift perturbations (CSP) between apo and holo NCS could further validate the accuracy of the AF-QM/MM approach. Among the hydrogen atoms in the binding pocket, H15, H31 of the ligand, HD2 of Leu45 and HB2 of Cys37 are significantly influenced by the ring current effect, where they are close to the aromatic rings in the native holo structure (see Figure 5). As a result, large upfield shifts upon ligand binding were observed for those atoms. The experimental CSPs of those four atoms (namely, H15, H31, Leu45:HD2, and Cys37:HB2) are −0.93, −0.86, −1.15, and −0.72 ppm, respectively, while the AF-QM/MM results of them are −0.45, −0.34, −1.25, and −0.71 ppm. The results show that large chemical shift perturbations between apo and holo protein-ligand systems could be accurately predicted by the AF-QM/MM approach.

### Performance of the CS_score_ scoring function

**Figure 6:**
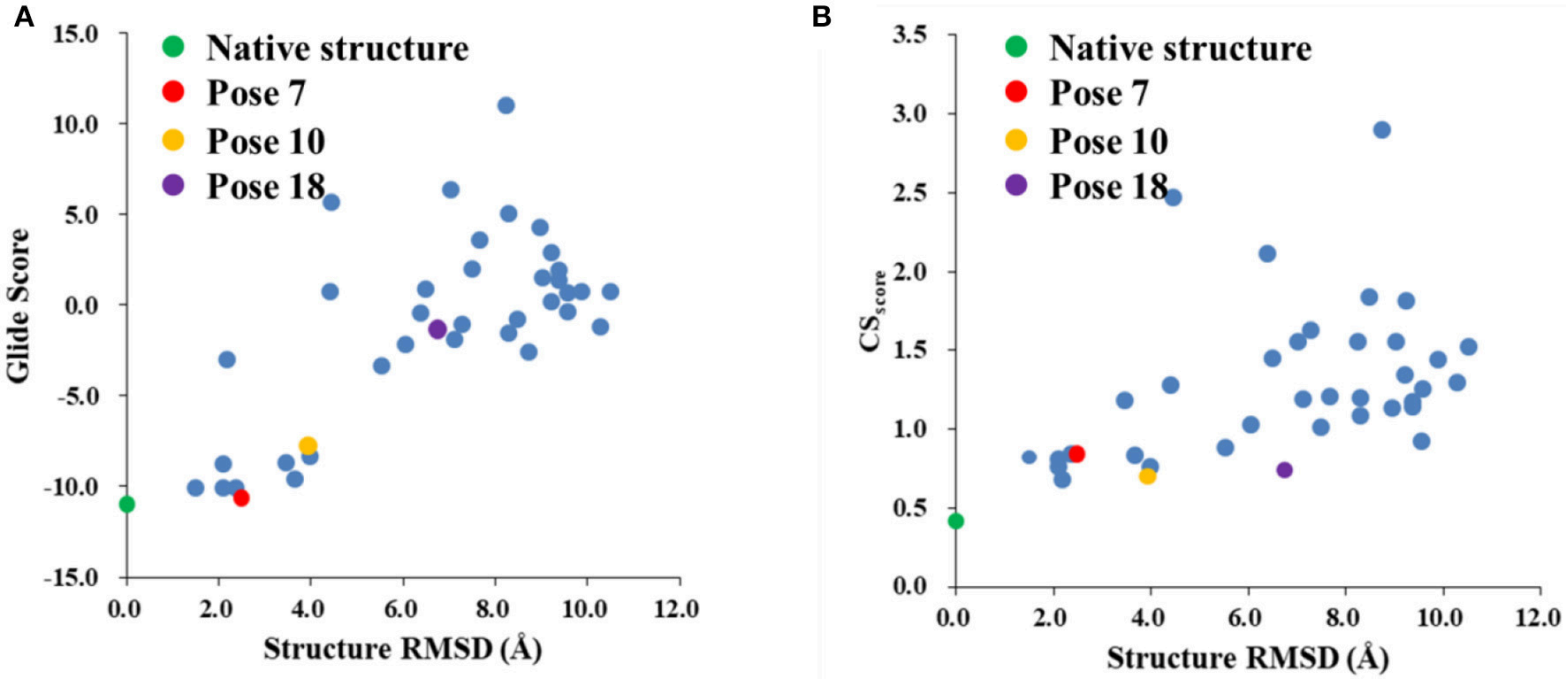
The Glide **(A)** and CSscore **(B)** scores with different structural RMSDs of the chromophore in the holo NCS. The green dot represents the score of the binding pose in the X-ray structure of the holo NCS. The red, yellow and violet dots denote the docking poses 7, 10 and 18, respectively.

The Glide scores of the 38 docked decoys and the native holo NCS are shown in Figure 6. The energy based scoring function is capable of distinguishing the experimental structure from the docked poses whose structural RMSDs are larger than 4 Å with reference to the native pose. However, for the docked poses whose RMSDs are between 2 and 4 Å, the Glide scores of them are sometimes very close to the experimental structure (see Pose 7 in Figure 6A). In contrast, the CS_score_ is easier to discriminate the experimental structure from the decoy sets whose structural RMSDs are around 2 Å. This is mainly due to that chemical shifts are quite sensitive to the local chemical environment at the binding site. In CS_score_, protons in ligand and the selected hydrogen atoms in protein residues serve as molecular probes to detect the binding environment. When the protons have different close contacts between the native and decoy structures, such as the interactions with aromatic rings or hydrogen bonding, the calculated chemical shift of certain protons may have substantial deviations between different binding modes. Therefore, the change of NMR chemical shifts of protons could clearly reflect the corresponding binding interactions between the protein and ligand.

**Figure 7:**
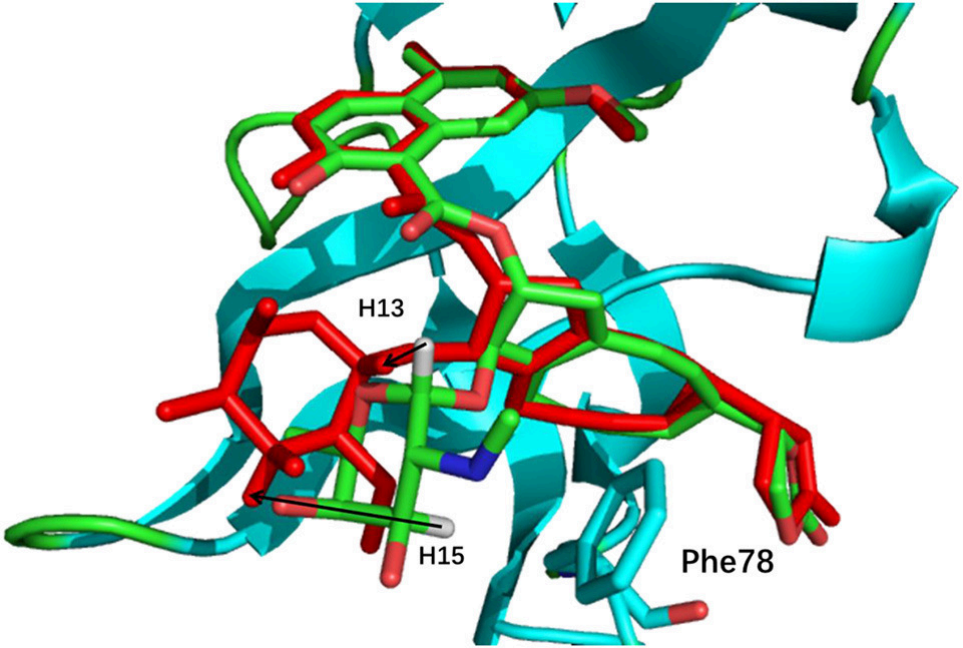
The comparison between the binding structure of Pose 7 and the native binding pose. The green stick model denotes the native binding pose, and the red stick model denotes the binding structure of Pose 7 from Gilde docking program. The aminosugar group in the chromophore was displaced in the docking pose 7 as compared to the experimental structure.

The comparison of calculated chemical shifts between the native and docking poses could probe the structural changes of ligand binding poses among them. Figure 7 shows that, for the positions of the naphthoate group and enediyne ring in Pose 7, the chromophore is very close to the native binding structure. However, the aminosugar group is pointing to a different direction, which results in the large chemical shifts deviation for H13 and H15 on the ligand (see Table 1).

**Table 1:** The comparison between the experimental and calculated chemical shifts (in ppm).

	**Experiment**	**Native**	**Pose 7**
LIG:H13	5.98	5.87	4.45
LIG:H15	3.42	3.12	4.31

*“Native” and “Pose 7” denote the calculated chemical shifts for the native binding pose and the docking pose of Pose 7, respectively*.

The weakness of CS_score_ is that for decoys with larger structural RMSDs, the chemical shift based scoring function might be not as efficient as those with low structural RMSDs. In high structural RMSD range, some ligand poses might be close to the apo state (fewer interactions with the protein), and the CS_score_ score for the apo state of chromophore is 0.60. Therefore, the rankings of those poses are not very sensitive to the structural RMSDs using CS_score_. The example cases for poses 10 and 18 will be discussed in Section Improvement of the hybrid CS_Gscore_ scoring function.

### Improvement of the hybrid CS_Gscore_ scoring function

**Figure 8:**
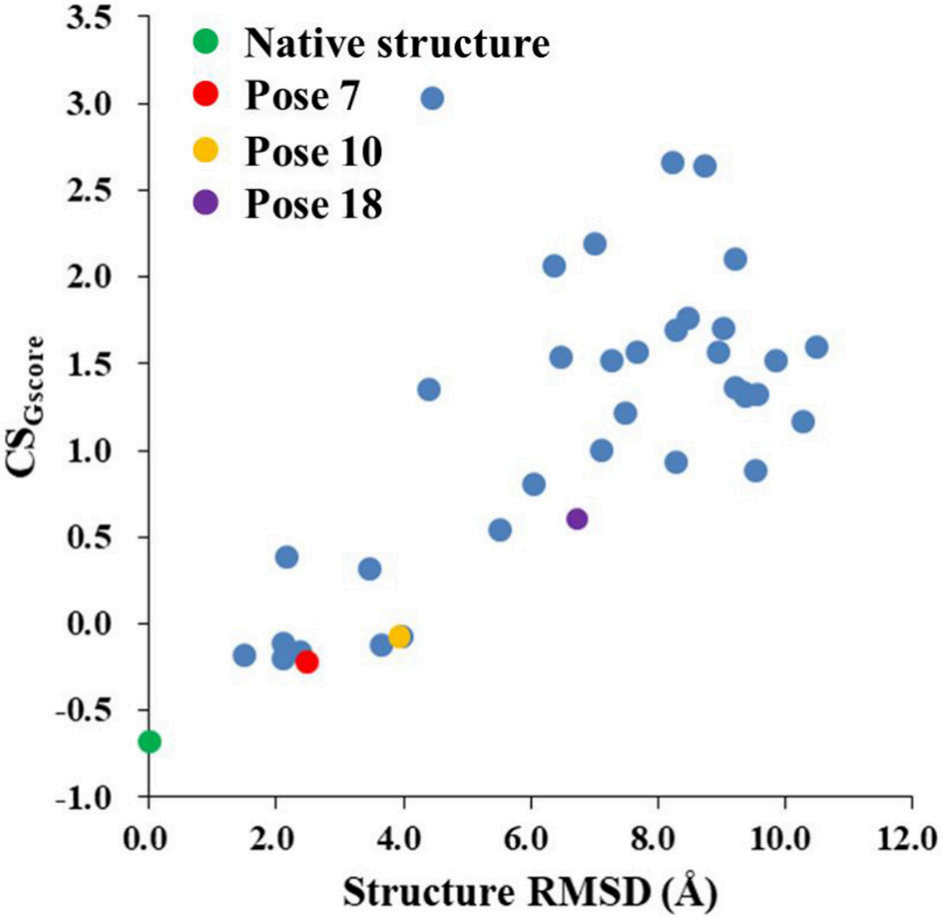
The rankings of the native (green) and docking structures (red: pose 7; yellow: pose 10; violet: pose 18; and blue: other docked poses, predicted by Glide) calculated by CS_Gscore_.

Figure 8 shows that the CS_Gscore_ score is capable of differentiating the experimental structure from the decoys for the protein-ligand complex. In this work, the weighting factor α in Equation (2) was to set to 0.1 to make the CS_score_ and Glide scores on the same scale. As shown in Figure 8, for poses whose structural RMSDs are around 2–4 Å, the CS_score_ score from NMR chemical shifts dominates the scoring function. Even though the Glide score for the decoy structures are close to the experimental structure (see Figure 6B), the CS_score_ could discriminate the experimental structure from the decoys, resulting that the CS_Gscore_ (combination of CS_score_ and Glide scores) ranked the native binding pose clearly as the most favorable structure. On the other hand, for decoy poses whose structural RMSDs are larger than 4 Å, the energy based scoring function (Glide score) has the major impact on the CS_Gscore_, which makes the decoys with large structural RMSDs deviates more from the experimental structure as compared to the CS_score_ score (Figure 6B).

**Figure 9:**
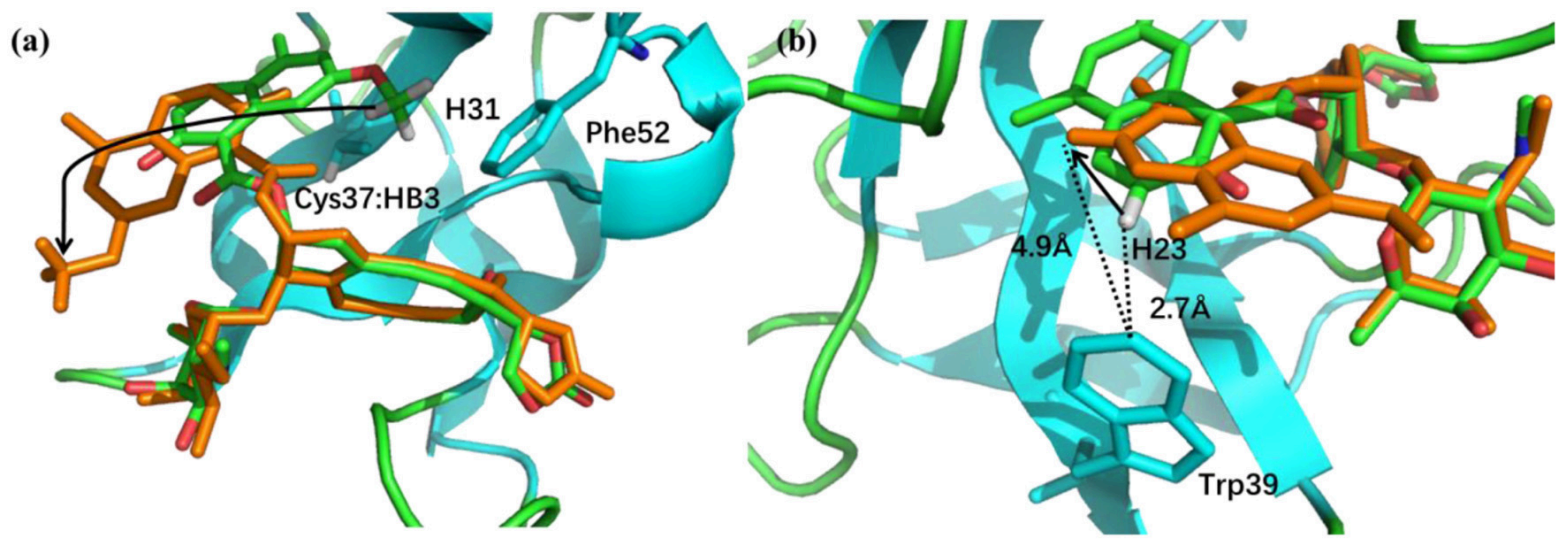
**(a)** The binding structures of pose 10 (orange) and the native pose (green). The flip of the naphthoate group made H31 and Cys37:HB3 to move away from the aromatic ring region, which will cause upfield chemical shift. **(b)** For pose 10, H23 moves away from the aromatic ring region, which will cause downfield chemical shift.

The pose 10 was previously scored low in CS_score_, whose chemical shift RMSD is 0.70 ppm between the calculated and experimental data. The naphthoate group of the ligand was flipped in the docking structure, while locations of the other regions of the chromophore are similar to the native binding pose (see Figure 9). On the naphthoate group, H23 has the largest chemical shift deviation between the native binding structure and pose 10. In the native binding pose, H23 is beside the aromatic ring, which causes downfield chemical shift on H23. While in Pose 10, the H23 atom moves away from the indole ring of Trp39, resulting that its calculated chemical shift is much lower than that of the experimental structure (see Table 2). Furthermore, Figure 9a shows that in Pose 10, H31 moves away from the phenyl ring of Phe52, and the corresponding chemical shielding decreased, resulting in higher NMR chemical shift. Furthermore, because the aromatic ring position of the naphthoate group moved in the docking structure, ^1^H chemical shift on surrounding protein residues, such as Cys37:HB3, also changed significantly. Meanwhile, since the naphthoate ring strongly influenced the ^1^H chemical shifts from the unbound to bound states, straying away of the naphthoate group in Pose 10 caused that the chemical shifts are less affected upon ligand binding, which results in slightly higher CS_score_ score (see Figure 6B, but other groups are close to the native state). Considering the physical non-bonded interactions between NCS and chromophore, as the naphthoate group stays deep inside the binding pocket in the native state, which contributes most to the NCS-chromophore binding energy. Therefore, the structural deformation in pose 10 caused that the interaction energy between them became weaker, and the corresponding Glide score gave the lower rank. In the hybrid scoring function CS_Gscore_, the ranking of pose 10 has a clear separation from the experimental structure, because it incorporates both the NMR chemical shift deviations from the experimental data and the physical interaction energy between the protein and ligand.

**Table 2:** Comparison between the experimental and calculated chemical shifts on the native binding model and pose 10 (in ppm).

	**Experiment**	**Native**	**Pose 10**
LIG:H23	7.40	7.91	6.14
LIG:H31	2.96	3.58	4.92
Cys37:HB3	2.17	2.08	3.19

**Figure 10:**
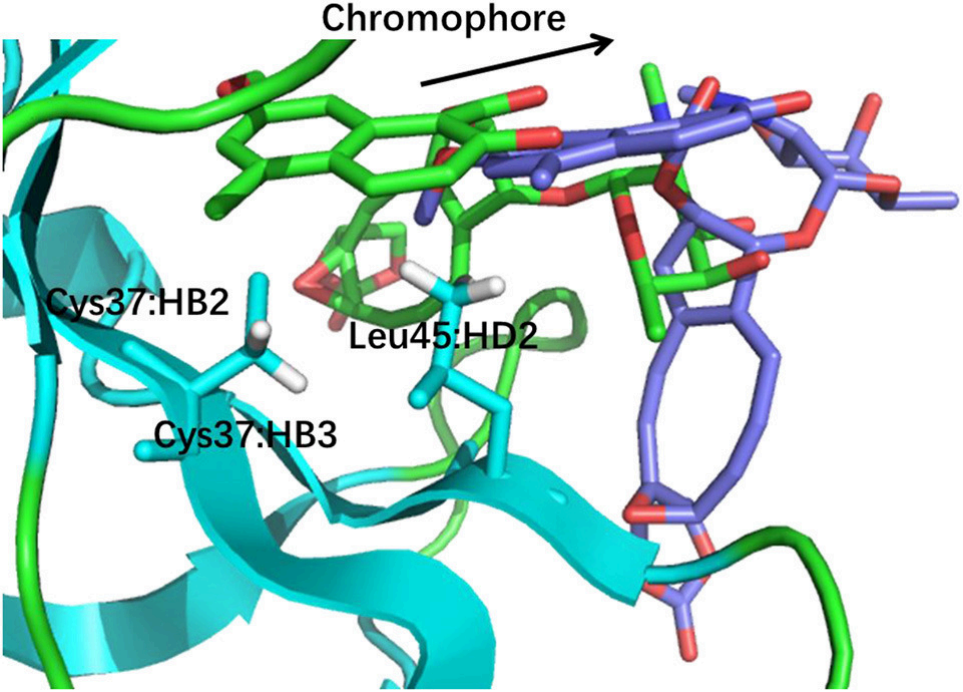
The structures of pose 18 (violet) and the native state (green). The chromophore in pose 18 shifted away from the position of the native state, making that Cys37:HB2, Cys37:HB3, and Leu45:HD2 move away from the aromatic rings of the chromophore.

The docking pose 18 is also the case that the energy function is more important than the CS_score_ in ranking the docking poses. Figure 10 shows that the ligand position of pose 18 almost translated to the direction away from the binding pocket. As the naphthoate ring moved away, the chemical shieldings of Cys37:HB2, Cys37:HB3 and Leu45:HD2 decreased as compared to the native state (see Table 3). The ligand pose 18 is closer to the apo state, which resulted in high CS_score_ score, but the interaction energy between the chromophore and NCS would be obviously weaker than that of the native state, and the Glide score for the pose 18 is substantially higher than the native binding pose (see Figure 6A). Therefore, in the hybrid scoring function CS_Gscore_, the ranking of pose 18 is clearly lower than the native state, which correctly reflects that the rankings decrease as the structural RMSDs become larger.

**Table 3:** Comparison between the experimental and calculated chemical shifts on the native state and the docking pose 18 (in ppm).

	**Experiment**	**Native**	**Pose 18**
Cys37:HB2	1.98	1.52	2.60
Cys37:HB3	2.17	2.08	2.82
Leu45:HD2	−0.90	−1.25	0.69

## Conclusion

In this work, we applied the automated fragmentation method for QM/MM calculation of NMR chemical shifts for protein-ligand binding complexes. In the AF-QM/MM approach, the atomic NMR chemical shifts were obtained by dividing the protein automatically into residue-centric fragments. In order to reduce the computational cost for the ligand, the chromophore that contains 81 atoms was also divided into three smaller fragments to make the QM size for ligand calculation comparable to the protein fragments. The AF-QM/MM approach with the implicit solvation treatment is computationally efficient and linear-scaling with a low pre-factor. Moreover, the approach is massively parallel and can be applied to routinely calculate the *ab initio* NMR chemical shifts for protein-ligand complexes of any size.

The ^1^H chemical shifts calculated by the AF-QM/MM approach at the DFT level are in good agreement with large-sized system calculation, where the entire ligand and its buffer region are treated by QM, and the remaining atoms of the protein are described by background charges. The MUE between AF-QM/MM and large-sized system calculation is 0.046 ppm. Furthermore, the MUEs between calculated and experimental ^1^H chemical shifts in the binding pocket of apo and holo NCS are 0.45 and 0.44 ppm, respectively. Our results demonstrate that the AF-QM/MM approach is capable of reproducing the large-sized system *ab initio* calculations of NMR chemical shifts for protein-ligand complexes, and the calculated chemical shifts are in good agreement with the experimental results.

The results of CS_score_ scores show that chemical shifts could be utilized as molecular probes to detect the binding conformation of the protein-ligand complex. The experimental structure has the clear leading score as compared to the decoy binding poses. By investigating the CSP patterns of decoy structures, the position changes of the ligand could be detected by variations of chemical shifts in different local chemical environment.

In this study, we further proposed the hybrid scoring function CS_Gscore_ which combines CS_score_ and the energy-based scoring function of Glide score. The hybrid CS_Gscore_ scoring function can help to distinguish the native ligand structure from the decoy docking poses. CS_Gscore_ can also clearly separate the scores of decoy structures, which have significantly large structural RMSD values and give relatively low CS_score_ scores, from the native docking pose. The CS_Gscore_ incorporates both the experimental NMR chemical shift information and the energy-based scoring method, which could better determine the binding site structure of the protein-ligand complex. Therefore, the AF-QM/MM approach provides an accurate and efficient platform for protein-ligand binding structure prediction based on NMR derived information.

## Author contributions

XH designed research; XJ, TZ, and XH performed research; XJ, TZ, JZ, and XH analyzed data; and XJ and XH wrote the paper.

### Conflict of interest statement

The authors declare that the research was conducted in the absence of any commercial or financial relationships that could be construed as a potential conflict of interest.
