# Supplementary material for: Automated Fragmentation QM/MM Calculation of NMR Chemical Shifts for Protein-Ligand Complexes
Source: Front Chem. 2018 May 8;6:150. doi: 10.3389/fchem.2018.00150 (PMC5952040; doi:10.3389/fchem.2018.00150)
Supplement: Supplementary file 1 [file Table_1.PDF]

Supplementary materials for

Automated Fragmentation QM/MM Calculation of NMR Chemical Shifts for Protein-Ligand Complexes

Xinsheng Jin[1], Tong Zhu[1,3], John Z.H. Zhang[1,3,4] and Xiao He[1,2,3]*

[1]State Key Laboratory of Precision Spectroscopy, School of Chemistry and Molecular Engineering, Shanghai Engineering Research Center of Molecular Therapeutics and New Drug Development, East China Normal University, Shanghai, 200062, China

[2]National Engineering Research Centre for Nanotechnology, Shanghai, 200241, China

[3]NYU-ECNU Center for Computational Chemistry at NYU Shanghai, Shanghai, 200062, China

[4]Department of Chemistry, New York University, NY, NY 10003, US

[*]Corresponding author: xiaohe@phy.ecnu.edu.cn

Table S1. The experiment chemical shifts (in ppm) for apo and holo NCS. The data of apo NCS is obtained from Mohanty et al.'s work (Mohanty et al., 1994), and chemical shifts of holo NCS is downloaded from BMRB (code: 5969).

res No.	res name	atom name	holo_exp	apo_exp	CSP
33	ASP	HA	5.22	5.16	0.06
33	ASP	HB2	2.13	2.19	-0.06
33	ASP	HB3	2.57	2.57	0.00
34	VAL	HA	4.94	4.88	0.06
34	VAL	HB	1.91	1.98	-0.07
34	VAL	HG11	0.85	0.79	0.06
34	VAL	HG12	0.85	0.79	0.06
34	VAL	HG13	0.85	0.79	0.06
34	VAL	HG21	0.91	0.81	0.10
34	VAL	HG22	0.91	0.81	0.10
34	VAL	HG23	0.91	0.81	0.10
35	GLY	HA2	3.84	3.33	0.51
35	GLY	HA3	4.94	4.59	0.35
36	GLN	HA	5.77	5.38	0.39
36	GLN	HB2	1.93	1.82	0.11
36	GLN	HB3	2.19	2.04	0.15
37	CYS	HA	5.56	5.69	-0.13
37	CYS	HB2	1.98	2.70	-0.72
37	CYS	HB3	2.17	2.93	-0.76
38	ALA	HA	4.66	4.99	-0.33
38	ALA	HB1	1.29	1.37	-0.08
38	ALA	HB2	1.29	1.37	-0.08
38	ALA	HB3	1.29	1.37	-0.08
39	TRP	HA	5.14	5.09	0.05
39	TRP	HB2	3.22	3.16	0.06
39	TRP	HB3	3.22	3.31	-0.09
39	TRP	HD1	7.39	7.30	0.09
39	TRP	HE3	7.57	7.51	0.06
39	TRP	HZ3	6.79	6.86	-0.07
39	TRP	HH2	7.34	7.30	0.04
39	TRP	HZ2	7.78	7.57	0.21
43	GLY	HA2	4.12	3.97	0.15
43	GLY	HA3	4.36	4.26	0.10
44	VAL	HA	4.4	4.39	0.01
44	VAL	HB	1.78	1.77	0.01
44	VAL	HG11	0.69	0.72	-0.03
44	VAL	HG12	0.69	0.72	-0.03
44	VAL	HG13	0.69	0.72	-0.03

44	VAL	HG21	0.97	0.92	0.05
44	VAL	HG22	0.97	0.92	0.05
44	VAL	HG23	0.97	0.92	0.05
45	LEU	HA	4.12	4.55	-0.43
45	LEU	HB2	-0.05	0.81	-0.86
45	LEU	HB3	-0.02	1.24	-1.26
45	LEU	HG	0.44	0.80	-0.36
45	LEU	HD11	-1.29	-0.14	-1.15
45	LEU	HD12	-1.29	-0.14	-1.15
45	LEU	HD13	-1.29	-0.14	-1.15
45	LEU	HD21	-0.90	-0.02	-0.88
45	LEU	HD22	-0.90	-0.02	-0.88
45	LEU	HD23	-0.90	-0.02	-0.88
46	ALA	HA	5.21	4.95	0.26
46	ALA	HB1	0.92	0.96	-0.04
46	ALA	HB2	0.92	0.96	-0.04
46	ALA	HB3	0.92	0.96	-0.04
47	CYS	HA	5.56	5.70	-0.14
47	CYS	HB2	3.10	3.19	-0.09
47	CYS	HB3	3.49	3.47	0.02
48	ASN	HA	5.74	5.53	0.21
48	ASN	HB2	2.52	1.34	1.18
48	ASN	HB3	2.55	2.46	0.09
52	PHE	HA	5.46	5.22	0.24
52	PHE	HB2	3.46	2.38	1.08
52	PHE	HB3	3.60	3.34	0.26
52	PHE	HD1	7.67	7.45	0.22
52	PHE	HE1	7.39	7.14	0.25
52	PHE	HZ	7.25	7.05	0.20
53	SER	HA	5.12	5.01	0.11
53	SER	HB2	3.89	3.82	0.07
53	SER	HB3	4.12	3.98	0.14
67	LEU	HA	4.91	4.74	0.17
67	LEU	HB2	1.68	1.22	0.46
67	LEU	HB3	1.68	1.22	0.46
67	LEU	HG	1.54	1.50	0.04
67	LEU	HD11	1.28	1.07	0.21
67	LEU	HD12	1.28	1.07	0.21
67	LEU	HD13	1.28	1.07	0.21
67	LEU	HD21	0.88	0.69	0.19
67	LEU	HD22	0.88	0.69	0.19
67	LEU	HD23	0.88	0.69	0.19
76	PHE	HA	5.50	5.43	0.07

76	PHE	HB2	2.63	2.78	-0.15
76	PHE	HB3	3.13	3.04	0.09
76	PHE	HD1	7.46	7.43	0.03
77	LEU	HA	5.45	4.95	0.50
77	LEU	HB2	2.00	1.99	0.01
77	LEU	HB3	2.25	2.25	0.00
77	LEU	HG	1.84	1.88	-0.04
77	LEU	HD11	0.90	0.88	0.02
77	LEU	HD12	0.90	0.88	0.02
77	LEU	HD13	0.90	0.88	0.02
77	LEU	HD21	1.27	1.16	0.11
77	LEU	HD22	1.27	1.16	0.11
77	LEU	HD23	1.27	1.16	0.11
78	PHE	HA	4.20	4.39	-0.19
78	PHE	HB2	3.32	3.29	0.03
78	PHE	HB3	3.40	3.36	0.04
78	PHE	HD1	7.44	7.41	0.03
78	PHE	HE1	7.66	7.41	0.25
79	ASP	HA	4.36	4.47	-0.11
79	ASP	HB2	2.13	2.41	-0.28
79	ASP	HB3	3.12	3.13	-0.01
80	GLY	HA2	4.16	3.78	0.38
80	GLY	HA3	4.31	4.49	-0.18
81	THR	HA	4.15	4.20	-0.05
81	THR	HB	4.20	4.20	0.00
81	THR	HG21	1.28	1.31	-0.03
81	THR	HG22	1.28	1.31	-0.03
81	THR	HG23	1.28	1.31	-0.03
93	CYS	HA	5.02	5.38	-0.36
93	CYS	HB2	3.33	3.34	-0.01
93	CYS	HB3	4.08	4.02	0.06
94	GLN	HA	5.57	5.57	0.00
94	GLN	HB2	1.88	1.52	0.36
94	GLN	HB3	2.02	2.18	-0.16
94	GLN	HG2	2.21	1.94	0.27
94	GLN	HG3	2.55	2.03	0.52
95	VAL	HA	5.51	4.91	0.60
95	VAL	HB	1.85	1.75	0.10
95	VAL	HG11	0.90	0.71	0.19
95	VAL	HG12	0.90	0.71	0.19
95	VAL	HG13	0.90	0.71	0.19
95	VAL	HG21	1.03	0.81	0.22
95	VAL	HG22	1.03	0.81	0.22

95	VAL	HG23	1.03	0.81	0.22
96	GLY	HA2	3.40	3.88	-0.48
96	GLY	HA3	3.81	4.36	-0.55
97	LEU	HA	5.52	5.47	0.05
97	LEU	HB2	0.85	1.18	-0.33
97	LEU	HB3	1.49	1.41	0.08
97	LEU	HG	1.33	1.49	-0.16
97	LEU	HD11	0.39	0.43	-0.04
97	LEU	HD12	0.39	0.43	-0.04
97	LEU	HD13	0.39	0.43	-0.04
97	LEU	HD21	0.50	0.59	-0.09
97	LEU	HD22	0.50	0.59	-0.09
97	LEU	HD23	0.50	0.59	-0.09
98	SER	HA	4.93	5.20	-0.27
98	SER	HB2	3.52	3.62	-0.10
98	SER	HB3	3.52	3.73	-0.21
99	ASP	HA	4.89	4.74	0.15
99	ASP	HB2	2.13	2.25	-0.12
99	ASP	HB3	3.04	2.96	0.08
100	ALA	HA	4.22	4.20	0.02
100	ALA	HB1	1.55	1.50	0.05
100	ALA	HB2	1.55	1.50	0.05
100	ALA	HB3	1.55	1.50	0.05
101	ALA	HA	4.45	4.55	-0.10
101	ALA	HB1	1.53	1.50	0.03
101	ALA	HB2	1.53	1.50	0.03
101	ALA	HB3	1.53	1.50	0.03
102	GLY	HA2	4.13	3.69	0.44
102	GLY	HA3	4.43	4.16	0.27
103	ASN	HA	5.17	5.09	0.08
103	ASN	HB2	2.86	2.84	0.02
103	ASN	HB3	3.10	3.17	-0.07
103	ASN	HD21	7.05	7.05	0.00
103	ASN	HD22	8.26	8.22	0.04
106	GLU	HA	4.37	4.30	0.07
106	GLU	HB2	2.12	2.13	-0.01
106	GLU	HB3	2.25	2.22	0.03
106	GLU	HG2	2.19	2.66	-0.47
106	GLU	HG3	2.19	2.66	-0.47
107	GLY	HA2	4.09	3.57	0.52
107	GLY	HA3	4.34	4.18	0.16
108	VAL	HA	4.32	4.26	0.06
108	VAL	HB	2.07	1.99	0.08

108	VAL	HG11	0.99	0.98	0.01
108	VAL	HG12	0.99	0.98	0.01
108	VAL	HG13	0.99	0.98	0.01
108	VAL	HG21	1.12	1.13	-0.01
108	VAL	HG22	1.12	1.13	-0.01
108	VAL	HG23	1.12	1.13	-0.01

Table S2. The structural RMSD of the ligand (with reference to the experimental binding structure), CS_{score}, Glide score and CS_{Gscore} for different binding poses. Pose 1 is the experimental binding structure.

Pose	Structural RMSD(Å)	CS_{score}	Glide score	CS_{Gscore}
1	0.00	0.42	-10.96	-0.68
2	1.50	0.82	-10.08	-0.19
3	2.18	0.68	-2.99	0.38
4	2.11	0.81	-10.11	-0.20
5	2.11	0.76	-8.77	-0.12
6	2.39	0.84	-10.09	-0.17
7	2.48	0.84	-10.63	-0.22
8	3.48	1.18	-8.68	0.31
9	3.67	0.83	-9.63	-0.13
10	3.93	0.70	-7.75	-0.07
11	3.99	0.76	-8.34	-0.08
12	4.42	1.28	0.69	1.35
13	4.46	2.46	5.66	3.03
14	5.54	0.88	-3.36	0.54
15	6.39	2.11	-0.48	2.06
16	6.07	1.02	-2.22	0.80
17	6.49	1.45	0.85	1.53
18	6.74	0.74	-1.33	0.61
19	7.04	1.55	6.34	2.19
20	7.29	1.62	-1.05	1.52
21	7.13	1.18	-1.92	0.99
22	7.68	1.20	3.58	1.56
23	7.50	1.01	1.97	1.21
24	8.50	1.83	-0.77	1.76
25	8.30	1.08	-1.57	0.92
26	8.31	1.19	5.00	1.69
27	8.24	1.55	10.98	2.65
28	8.97	1.13	4.30	1.56
29	9.05	1.55	1.47	1.70
30	8.75	2.90	-2.64	2.63
31	9.38	1.14	1.91	1.33
32	9.24	1.81	2.87	2.10
33	9.57	0.92	-0.36	0.88
34	9.23	1.34	0.20	1.36
35	9.39	1.17	1.35	1.31
36	9.59	1.25	0.66	1.32
37	10.52	1.52	0.74	1.59
38	9.89	1.43	0.75	1.51

39	10.30	1.29	-1.24	1.17

References:

1) Mohanty, S., Sieker, L.C., and Drobny, G.P. (1994). Sequential ^{1}H NMR assignment of the complex of aponeocarzinostatin with ethidium bromide and investigation of protein-drug interactions in the chromophore binding site. *Biochemistry* 33(35)**,** 10579-10590. doi: 10.1021/bi00201a003.
